# Role of Vesicular Monoamine Transporter 2 Inhibitors in Tardive Dyskinesia Management

**DOI:** 10.7759/cureus.5471

**Published:** 2019-08-24

**Authors:** Venkatesh Sreeram, Shanila Shagufta, Faisal Kagadkar

**Affiliations:** 1 Psychiatry, Harlem Hospital Center, New York, USA; 2 Psychiatry, Delaware Psychiatric Hospital, Corona, USA; 3 Psychiatry, Rutgers New Jersey Medical School, Newark, USA

**Keywords:** tardive dyskinesia, schizophrenia, valbenazine, deutetrabenazine, vmat2 inhibitors, tetrabenazine, psychosis, atypical antipsychotics, aims, neuroleptic

## Abstract

Tardive dyskinesia (TD) is a distressing and disabling movement disorder that occurs with the use of chronic neuroleptic medications. TD is defined as involuntary athetoid or choreiform movements of head, trunk or limbs. Tongue, lower face, jaw, and extremities are commonly involved but pharyngeal, diaphragmatic, or truncal muscles are also sometimes involved affecting breathing, swallowing, speech, posture, gait, and mobility of an individual.

TD is a debilitating movement disorder that requires timely intervention. Subtle tongue movements, tic-like facial movements or increased blink frequency could be some of the initial manifestations of TD. Our article is focused on the new advents in treating TD, their efficacy, and tolerability with emphasizing their side effect profile. The implication of a genetic marker vesicular monoamine transporter 2 (VMAT2), helped in investigating VMAT2 inhibitors for alleviating TD. Among the modalities tested, only VMAT2 inhibitors reported efficacy. However, the outcome of long-term use and its side effect profile can only be determined with longer studies utilizing large set data. More clinical trials are required to explore individual drug efficacy and their long-term adverse effects.

We aim to provide an overview of TD management, illustrating the priority of VMAT2 inhibitors and to determine the importance of selecting an optimal medication. A search through PubMed with terms "Tardive dyskinesia" and "VMAT2 inhibitors" was carried out. Several treatment modalities were tested to control the symptoms of TD with limited benefit. However, VMAT2 inhibitors showed improvement in the Abnormal Involuntary Movement Scale (AIMS) rating scale for TD. Valbenazine and deutetrabenazine (d-TBZ) were recently approved by the Food and Drug Administration (FDA) for treating TD in adults.

## Introduction and background

Tardive dyskinesia (TD) is an alarming movement disorder that occurs due to dopamine receptor blockers. The first case of TD was identified in 1957 as a permanent orofacial hyperkinetic movement. It was referred to as “paroxysmal dyskinesia” linked to neuroleptics [1]. TD was commonly seen with the chronic use of psychotropic medications. With the advent of second-generation antipsychotics (SGAs), or atypical antipsychotics, many providers expected the resolution of the potential side effects of conventional antipsychotics. Although SGAs reduced the incidence of side effects, including TD, they did not eliminate them [2].

TD is manifested with abnormal involuntary movements of the face and tongue, such as chewing, lip smacking, and tongue protrusion or thrust, along with choreiform movements of extremities. The Abnormal Involuntary Movement Scale (AIMS) is an assessment tool commonly used to rate dyskinesia [3]. The pathophysiology of TD is poorly understood, with the majority of studies reporting it as a result of a blockage of dopamine receptors for a longer duration. Furthermore, genetic markers have been tested in causing TD [4].

TD shows irreversibility with the discontinuation of the antipsychotic agent in most cases, which led to the hypothesis that it is a disorder of neuroplasticity. D2 receptor hypersensitivity in the striatum leads to D2 antagonism, but the continuous blockade results in the synaptic plasticity of glutaminergic interneurons, to the extent of no symptomatic reversal after discontinuation of antipsychotics. This hypothesis supported the evidence of the benefit of glutaminergic-based medications and pallidal deep brain stimulation (DBS) in TD [5].

Psychiatric patients are often managed with lifelong psychotropic medications, which predispose them to develop the debilitating effects of TD. The remission rate of TD after discontinuation of an antipsychotic medication is 2% [6]. Several treatment modalities were tested for the treatment of TD, with limited benefit. They include amantadine, propranolol, piracetam, and levetiracetam, acetazolamide, bromocriptine, thiamine, zolpidem, vitamin E, vitamin B6, calcium channel blockers, selegiline, Gingko biloba, melatonin, buspirone, botulinum toxin type A, electroconvulsive therapy (ECT), switching to clozapine and DBS [7]. However, these modalities showed insufficient evidence to support or refute using them in TD. D-serine is a naturally occurring amino acid that acts in vivo as a positive modulator on the glycine site. It is associated with the glutamatergic N-methyl-d-aspartate (NMDA) receptor and regulates movement. It is currently being evaluated for use in TD [8].

The recent advances have allowed for a better understanding of the pathophysiology and genetic component involved in TD. Vesicular monoamine transporter 2 (VMAT2) was identified as a genetic marker. VMAT2 is responsible for monoamine transport across synaptic vesicles. VMAT transporters utilize the low pH of the lumen vesicle to segregate dopamine, norepinephrine, and serotonin into a synaptic vesicle. VMAT1 is found peripherally, while VMAT2 is seen in the brain. VMAT2 inhibitors are involved in interrupting the transport and degradation of monoamines by monoamine oxidase, leading to the depletion of monoamines in the synaptic pool. As TD is hypothesized to be caused by postsynaptic dopamine receptor hypersensitivity that leads to synaptic plasticity, the implication of VMAT2 in the pathophysiology of TD has been confirmed. Hence, VMAT2 receptor inhibition led to a selective, reversible decrease in monoamines and made it a potent treatment for TD [8-9]. It also helped in leading to a targeted treatment using the novel drugs valbenazine and deutetrabenazine (d-TBZ). This has provided clinicians and patients with promising treatment options and hope about the amelioration of TD’s symptoms. 

## Review

Tetrabenazine (TBZ) was one of the first VMAT2 inhibitors to be developed in the treatment of schizophrenia. In 2008, the Food and Drug Administration (FDA) approved TBZ for chorea in Huntington’s disease (HD) [8-9]. TBZ was only used as an off-label medication for Tourette’s disorder and TD. Valbenazineis also a VMAT2 inhibitor that inhibits irreversibility, in contrast to other VMAT2 inhibitors [10]. However, it was later discontinued for its low tolerance and potential adverse effect profile.

Valbenazine

Valbenazine is the purified form of the (+)-alpha isomer of TBZ. A placebo-controlled six-week clinical trial conducted by KINECT 3 in patients with moderate to severe TD reported improvement, with a significant difference in AIMS score at Week 6 for both study groups, which was evaluated by using the Clinical Global Impression-Tardive Dyskinesia (CGI-TD) scale outcome [11]. The FDA approved valbenazine as the first drug for the treatment of tardive dyskinesia in adults. Valbenazine is usually demonstrated as well tolerated and has a favorable pharmacokinetic profile with once-daily dosing. The common side effects documented are somnolence, headache, and urinary tract infections. However, none of these side effects led subjects to discontinue the medication. Moreover, valbenazine was not demonstrated to increase suicidal tendencies, including ideation and behavior, in patients with TD [12]. The one-year follow-up study data using valbenazine also suggested consistency in showing similar results. FDA labeling has contraindicated valbenazine use with substances that are potent inducers of CYP3A4 (which increases exposure to the active metabolite of valbenazine that causes adverse reactions) and MAO inhibitors (which increase the concentration of monoamines in synapses and thus increase adverse reactions) [13]. It is also not recommended for use in patients with prolonged QT, as it may increase the QT-interval.

Deutetrabenazine (d-TBZ)

D-TBZ is considered a selective VMAT2 inhibitor that contains deuterium. Deuterium is a compound that slows down the metabolism and prolongs the half-life; hence, fewer plasma concentration fluctuations were seen with d-TBZ. D-TBZ has demonstrated a reduction in neuropsychiatric symptoms in HD, such as depression, anxiety, and akathisia [14]. A randomized clinical trial reported efficacy with d-TBZ in TD [15]. In contrast to TBZ, d-TBZ exhibited infrequent dose fluctuations and withdrawals. It has also been shown to be safe when used with other antipsychotic medications. Another study with fixed dosing of d-TBZ reported 50% improvement in AIMS score than placebo in the study groups [16]. Since these two studies evaluated the efficacy and safety in TD, d-TBZ has been recently approved by the FDA in the United States. Also, d-TBZ is the first drug to be approved for both TD and HD for chorea. However, trials have reported lowering of mood and increased risk of suicidality with d-TBZ [17]. It is approved with a black-box warning of increasing the depression risk, with suicidal tendencies. Hence, further trials with a large sample size are warranted to investigate more adverse effects with prolonged use.

## Conclusions

VMAT2 inhibitors are shown to improve symptoms in individuals with TD. Valbenazine was the first VMAT2 inhibitor to get FDA approval for TD. In contrast to TBZ, valbenazine has been tolerated well with a once-daily dose and fewer side effects in short-term trials. The common side effects experienced were somnolence, fatigue, and headache that fluctuate along with a change in dose. Valbenazine has been shown to be effective in the management of TD with ease in tolerability and fewer adverse effects. D-TBZ is a novel VMAT2 inhibitor recently approved by the FDA in the treatment of TD. It has been found to be well tolerated with lower dose fluctuations and side effect profile. It has a similar side effect profile to other VMAT2 inhibitors, including akathisia, somnolence, fatigue, diarrhea, and depression. Moreover, d-TBZ allows the steady use of antipsychotic agents for underlying psychotic illness. With the approval of valbenazine and d-TBZ, the ideal medication can be studied by comparing their outcomes. Long-term benefits of both medications are required to be determined in future studies. Alternatively, other modalities, including DBS, are also being tested in resolving the symptoms of TD.

## References

[1] Schonecker M (1957). Paroxysmal dyskinesia as the effect of megaphen [Article in German]. Der Nervenarzt.

[2] Khouzam HR (2015). Identification and management of tardive dyskinesia: a case series and literature review. Postgrad Med.

[3] Guy W (1976). Abnormal Involuntary Movement Scale (AIMS); ECDEU Assessment Manual for Psychopharmacology. ECDEU assessment manual for psychopharmacology. 1976. Rockville, MD: US Dept of Health, Education and Welfare.534-7.

[4] Tsai HT, Caroff SN, Miller DD (2010). A candidate gene study of tardive dyskinesia in the CATIE schizophrenia trial. Am J Med Genet.

[5] Aquino CCH, Lang AE (2014). Tardive dyskinesia syndromes: current concepts. Parkinsonism Relat Disord.

[6] Vinuela A, Kang UJ (2014). Reversibility of tardive dyskinesia syndrome. Tremor Other Hyperkinet Mov.

[7] Meyer JM (2016). Forgotten but not gone: new developments in the understanding and treatment of tardive dyskinesia. CNS Spectrums.

[8] Witter DP, Holbert RC, Suryadevara U (2017). Pharmacotherapy for the treatment of tardive dyskinesia in schizophrenia patients. Expert Opin Pharmacother.

[9] Jankovic J (2016). Dopamine depleters in the treatment of hyperkinetic movement disorders. Expert Opin Pharmacother.

[10] Chen JJ, Ondo WG, Dashtipour K, Swope DM (2012). Tetrabenazine for the treatment of hyperkinetic movement disorders: a review of the literature. Clin Ther.

[11] Hauser RA, Factor SA, Marder SR (2017). KINECT 3: a phase 3 randomized, double-blind, placebo-controlled trial of valbenazine for tardive dyskinesia. Am J Psychiatry.

[12] Factor SA, Remington G, Comella CL (2017). The effects of valbenazine in participants with tardive dyskinesia: Results of the 1-year KINECT 3 extension study. J Clin Psychiat.

[13] Müller T (2015). Valbenazine granted breakthrough drug status for treating tardive dyskinesia. Expert Opin Inv Drug.

[14] Frank S, Testa CM, Stamler D (2016). Effect of deutetrabenazine on chorea among patients with Huntington disease: a randomized clinical trial. JAMA.

[15] Fernandez HH, Factor SA, Hauser RA (2017). Randomized controlled trial of deutetrabenazine for tardive dyskinesia: the ARM-TD study. Neurology.

[16] Anderson KE, Stamler D, Davis MD (2017). Deutetrabenazine for treatment of involuntary movements in patients with tardive dyskinesia (AIM-TD): a double-blind, randomised, placebo-controlled, phase 3 trial. Lancet Psychiatry.

[17] Kim AP, Baker DE, Levien TL (2018). VMAT2 inhibitors: new drugs for the treatment of tardive dyskinesia. Consult Pharm.

